# Mass Surveilance of *C. elegans*—Smartphone-Based DIY Microscope and Machine-Learning-Based Approach for Worm Detection

**DOI:** 10.3390/s19061468

**Published:** 2019-03-26

**Authors:** Julia Bornhorst, Eike Jannik Nustede, Sebastian Fudickar

**Affiliations:** 1 Department of Food Chemistry, Institute of Nutritional Science, University of Potsdam, Arthur-Scheunert-Allee 114-116, 14558 Nuthetal, Germany; bornhorst@uni-wuppertal.de; 2 Food Chemistry, Faculty of Mathematics and Natural Sciences, University of Wuppertal, Gaußstraße 20, 42119 Wuppertal, Germany; 3 Assistance Systems and Medical Device Technology, Department of Health Services Research, Faculty of Medicine and Health Sciences, Carl von Ossietzky University Oldenburg, Ammerlaender Heerstr. 114-118, 26129 Oldenburg, Germany; eike.jannik.nustede@uni-oldenburg.de

**Keywords:** *Caenorhabditis elegans*, machine learning, smartphone, microscope, SVM, HOG

## Abstract

The nematode *Caenorhabditis elegans (C. elegans)* is often used as an alternative animal model due to several advantages such as morphological changes that can be seen directly under a microscope. Limitations of the model include the usage of expensive and cumbersome microscopes, and restrictions of the comprehensive use of *C. elegans* for toxicological trials. With the general applicability of the detection of *C. elegans* from microscope images via machine learning, as well as of smartphone-based microscopes, this article investigates the suitability of smartphone-based microscopy to detect *C. elegans* in a complete Petri dish. Thereby, the article introduces a smartphone-based microscope (including optics, lighting, and housing) for monitoring *C. elegans* and the corresponding classification via a trained Histogram of Oriented Gradients (HOG) feature-based Support Vector Machine for the automatic detection of *C. elegans*. Evaluation showed classification sensitivity of 0.90 and specificity of 0.85, and thereby confirms the general practicability of the chosen approach.

## 1. Introduction

For the more ethical use of animal experiments, the “3R” (reduction, refinement, and replacement) scientific concept is a guiding principle. Therefore, simpler organisms such as the threadworm *Caenorhabditis elegans (C. elegans)* are often used in toxicological, biomedical, and aging evaluations as a model system, since they offer several intrinsic advantages. Characteristics that have contributed to its utility as a model include easy and cheap maintenance, short life cycle (about 3.5 days at 20 °C), large brood size (≥250), well-developed genetics, and amenability to transgenesis [1]. Due to its transparency, morphological changes can be directly seen under a microscope (adult *C. elegans* are about the size of an eyelash (about 1 mm in length) [2]). Such changes can be caused by genetic defects, diseases, or other environmental influences, and are therefore very suitable for investigating toxicity and development. Since several core metabolic pathways are conserved between humans and *C. elegans*, it has become a well-established model for neurodegenerative diseases and metabolic disorders such as diabetes or obesity [3]. Additionally, the nematode is used in developmental biological research. Growth and developmental assays have been used to identify chemicals for potential developmental effects.

The detection and analysis of *C. elegans* is supported by software such as Worm Toolbox [4] or WorMachine [5,6], which enable the automated measurement of worm-movement parameters [7], and the classification of different worm types with defective molecules [8]. Concerning *C. elegans* analysis, there are also pure software solutions, e.g., by utilizing the ImageJ program with the Fiji Package [9,10].

In addition to the classification of different phenotypes, locomotion is a major focus of research, especially for neuronal studies. The locomotory patterns of the nematode are modulated by neurotransmitters like dopamine and serotonin. For MATLAB, there is a program that analyzes all common parameters [11]. Furthermore, a tracking microscope was developed that can analyze worm movement and its behavior based on LabVIEW [12]. These approaches for automated analysis are based on a common image-analysis approach: The worms are extracted from the background and then skeletonized [8]. The subsequent feature determination varies according to the selected parameters. The focus is often on tracking worm movement in video recordings. However, many of these programs are not fully automated, such as WormSizer [10], QuantWorm [13], or WormToolbox [4]. Currently, the software of Hakim et al., called WorMachine [5], represents the most enhanced approach: For the training of deep-learning classifiers, databases of *C. elegans* are generated by respective users. These databases are specialized on different morphological features according to the user’s wishes and goals. For analysis, binary classification with Support Vector Machines and more complex methods like Principal Component Analysis and T-distributed Stochastic Neighbor Embedding (t-SNE) have been implemented. However, existing solutions require the use of high-resolution images (and, thus, the availability) of a microscope. Even though such systems offer a wide range of imaging methods, such as bright- and dark-field microscopy, contrast methods (phase and interference contrast), or fluorescence microscopy, and thereby achieve good results for *C. elegans* detection, their significant acquisition costs limit their use for large-scale experiments.

However, even these software systems require the manual preselection of *C. elegans* in the microscope. The conventional manual observation of worms and the associated evaluation is time-consuming, labor-intensive, biased, and error-prone, thereby limiting the variety of experiments. Additionally, the high price of the required equipment for conventional screening as well as the software systems require cost-intensive equipment (both flow cytometer and microscopes). This strongly limits the use of *C. elegans* for toxicity trials, especially in emerging countries. Consequently, the automatic observation and analysis of *C. elegans* should be implemented for common low-price camera systems to achieve significant cost and labor savings, to ultimately enable the wider use of *C. elegans* for investigations, especially for high-throughput screening.

In this regard, the application of smartphones and machine-learning classifiers is a promising alternative due to the following reasons: The combined use of common smartphones with specific external accessories (including LED illumination sources and optical-magnification components) for smartphone-based microscopy systems has been shown to be well-suited to analyze biological samples of cells, microorganisms, and DNA 42 regarding variations in size, shape, brightness, or color [14]. Available smartphone-based microscopes cover a wide range of sensing capabilities, including microscopic imaging sensors, colorimetric sensors, electrochemical sensors, and electrochemiluminescence sensors [15]. Nowadays, the practicability of smartphone-based microscopic imaging sensors that combine smartphone-inherent image-processing capabilities with the (magnifying) optic systems of conventional microscopes to capture and analyse subcellular resolution images of objects in the microscopic and nanoscopic scale has been confirmed [16,17,18]. However, analysis of complete Petri dish scans with sufficient image resolution is still challenging regarding the trade-off between resolution and the field of view inherent to optical systems that are associated with smartphone optics [19]. In order to support automated examination of *C. elegans*, a complete scan of Petri dishes at once is essential. Here, the resulting image resolution of smartphone-camera sensors must achieve sufficient image resolution to accurately segment *C. elegans* while covering a complete Petri dish. However, the suitability of corresponding lower-resolution images to classify *C. elegans*, e.g., by applying machine learning, has not yet been investigated. Consequently, a system is proposed to replace the complex and expensive microscope for *C. elegans* studies, capture images of Petri dishes using a smartphone app, and automatically detect *C. elegans* in these images using a Support Vector Machine (SVM)-based classifier. Based on this system, the sensitivity to detect *C. elegans* was investigated.

## 2. Materials and Methods

**Figure 1:**
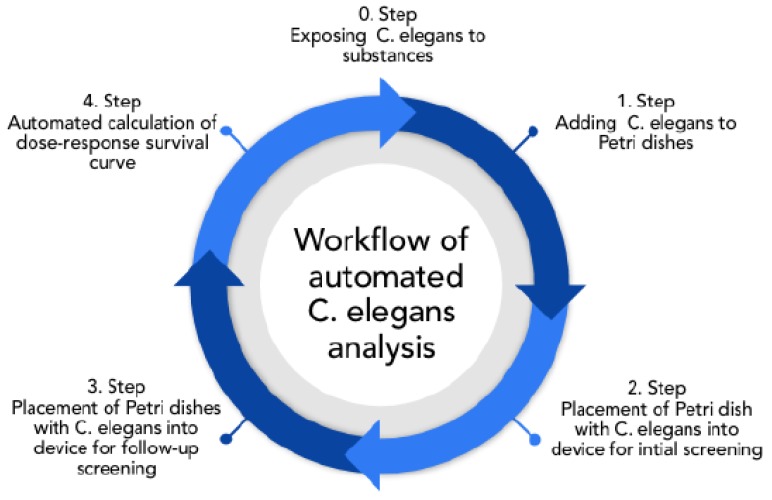
Intended workflow for automated *C. elegans* trials.

With the overall aim to propose an inexpensive smartphone-based microscope for the automated analysis of *C. elegans*, the following analyzing workflow was envisioned, as shown in Figure 1.

Initially, synchronized worms (all organisms of the same age) were exposed to the substance of interest. After treatment, worms were transferred to the OP50 spread NGM-plates and directly screened (Steps 1,2). After 24–48 h, the worms were again screened to identify dead individuals and obtain dose–response–survival curves (Steps 3,4). In the past, screening was done using a costly microscope and by detecting live and dead animals by eye [20,21]. Since about 50 animals needed to be counted per dish, with 3 independent dishes per concentration, manual counting is very time-consuming, taking several minutes per dish. Therefore, the intended technique overcomes this issue, as it enables quick scans that take less than 1 min per Petri dish.

Consequently, with an automatic procedure, it is possible to evaluate trials with just a few clicks, even during ongoing examinations to gain instant feedback on the suitability of the trial (in regard to the sufficiency of toxicity, and the quantity of samples and corresponding adjustments).

The intended automated calculation of the dose–response survival curve in the 4th step can be extended as desired (e.g., by calculating the worms’ average movement rate) after all data are recorded. Thereby, next to an increased throughput, additional measures can be applied.

### 2.1. Sensor Design

We developed the system as an Android application due to the low price and omnipresence of Android smartphones. Since the smartphones’ camera optics have insufficient magnification characteristics, an additional magnification lens had to be included aside of a Petri dish holder and the lighting system into the microscope. In addition, suitable image-processing and -classification algorithms had to be developed. Consequently, in regard to the system design, the following subtasks had to be realized, and are discussed in detail within the subsequent subsections:

- Design of microscope hardware to capture *C. elegans* with sufficient resolution and magnification.
- Design of a smartphone application to create and analyze worm images.
- Training a machine-learning classifier for the automatic recognition of *C. elegans*.

#### 2.1.1. Capture Hardware

For image capturing during the screening steps, the following hardware was developed that enables the use of standard smartphone lens systems to capture *C. elegans* on the complete Petri dish (diameter of 35 mm) with an image resolution that is sufficient for automated detection. In addition, the hardware assures standardized and stable imaging conditions (e.g., in terms of the illumination and positioning of the Petri dishes) by excluding external noise, and achieves the optimal illumination and positioning of the Petri dishes. During hardware design, it was intended to assure its applicability with various types of smartphones.

The concept of backlight microscopy, in which light sources face toward the camera lens and *C. elegans* attenuates the light beams, was applied due to the exclusion of reflections, which are potentially critical for classification accuracy.

**Figure 2:**
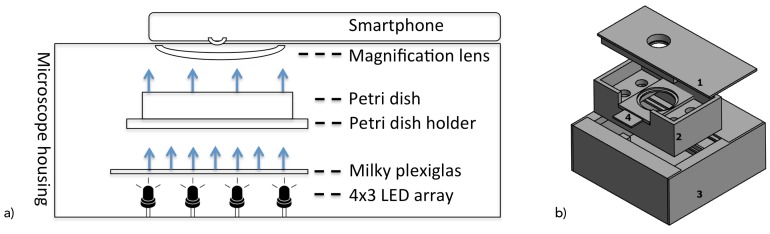
(**a**) Microscope casing as schematic indicating components. (**b**) Microscope casing consisting of (1) lid holding the smartphone and a 12× lens (in the cavity); (2) main carrier holding the lid within working distance; (3) light source; (4) removable holder for Petri dishes.

Consequently, as shown in Figure 2, the system consists of a 12 white light-emitting diode (LED) with a brightness of 2800 millicandela, and a 25° beam angle was applied in a 4 by 3 array as a light source that was placed below the Petri dishes. Furthermore, since LEDs emit a focused (spot) light beam, a 3 mm thick, 10 by 7.5 cm white opaque acrylic glass with 78% light transmission rate was placed at a distance of 2.7 cm to the LEDs, acting as a diffuser for scattering the light to assure a homogeneous illumination of the Petri dish in correspondence to Reference [22]. These exposure conditions and component distances were identified by visual inspection (under consideration of high contrast, equal lighting, and full coverage of the Petri dish) of the resulting raw images.

The major challenges in this setup were the optic requirements for the image acquisition. With the aim to integrate image acquisition into a smartphone app, the following two conditions of the smartphones’ camera hardware had to be concurrently fulfilled:

1. Camera viewing angles had to sufficiently cover a full Petri dish at a distance.
2. Camera image resolution and magnification had to be sufficient to detect worms and eventually derive additional quality parameters.

**Figure 3:**
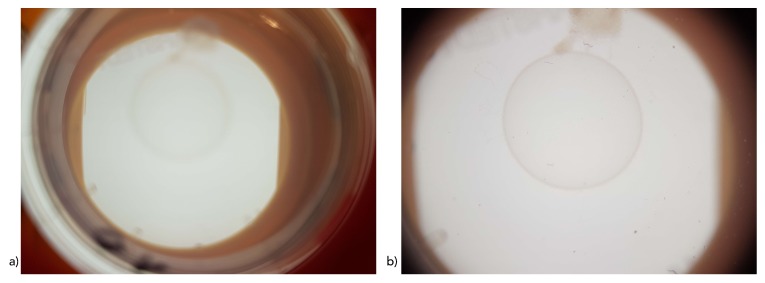
Corresponding example images (**a**) without magnification and (**b**) with magnification of *C. elegans* as captured with the system and a Samsung Galaxy S7 smartphone.

Common smartphone cameras (as integrated in the Galaxy S7, which was used in our evaluation) have a common resolution of 12 megapixels (MP) and an aperture of f/1.6 to f/2 (corresponding to an effective aperture of around f/11 to f/12) and an focal length of approximately 4.6 mm (correspondingly effective focal length of 26 mm) sufficient to capture adequate images. However, with current smartphone-camera systems not being designed to capture macroimages, smartphone optics are rather unsuitable for the intended purpose. Thus, lens systems were extended by a 12-fold magnification lens with a radius of 10.5 and 7 mm height (12× macrolens for smartphones by Apexel India). The system benefits from the fact that complex lens systems of current smartphones automatically try to adjust sharpness and to improve or compensate illumination by internal image-processing methods. Thus, a manual-focus setting as is required in traditional microscopes can be skipped, and only a magnifying lens is needed to magnify Petri dish images onto the smartphone camera. In order to adjust the focus for specific smartphone lenses, the working distance between the lenses and the Petri dish can be adjusted by a series of screws. Corresponding resulting images are shown in Figure 3.

**Figure 4:**
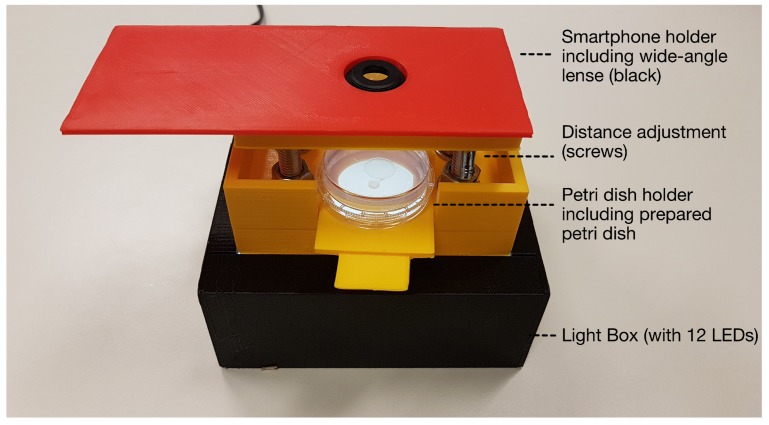
Developed microscope-prototype housing (screws were lifted for demonstration purposes, gap between smartphone holder and Petri dish holder is closed during operation).

In order to assure consistent recording conditions and obtain comparable images, the distance between all components was stabilized, and external (light) influences were excluded by the developed housing (shown in in Figure 4) that holds the lenses, smartphone, and Petri dish in the corresponding fixed positions. Housing had an overall height of 10.7 cm (11.9 × 12.2 cm form factor for the light-source box) with a 2 cm distance between acrylic glass and the Petri dish, Petri dish (bottom) to the macrolens of 2.6 cm.

In order to assure the accurate placement of the Petri dishes, a dish holder was designed to hold 35 mm Petri dishes within a corresponding carved position and, once fully slid into the system, places it on the visual sweet-spot focal plane.

#### 2.1.2. Smartphone Application for Capturing and Analysis

**Figure 5:**
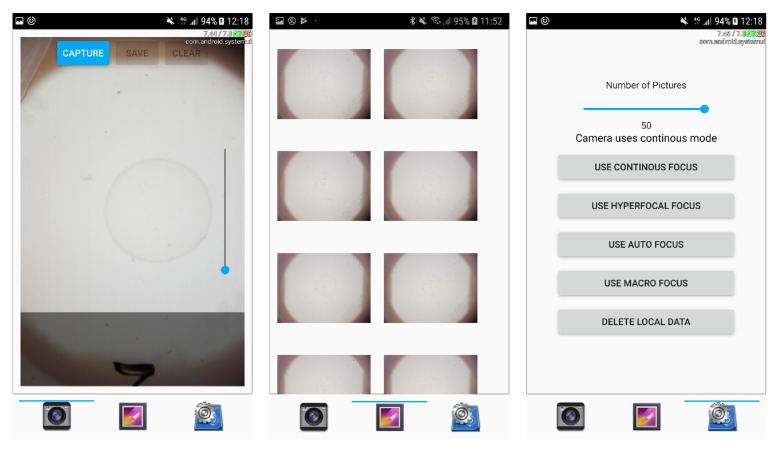
Smartphone application’s UI Components: image-capture dialog, gallery dialog, and settings dialog.

The smartphone application consists of a user interface for the capture, storage, and classification of images next to integrating image-processing and worm-detection functionality. The current application’s user interface (shown in Figure 5) consists of the following 3 dialogs:

- The image-capture dialog mainly shows the camera and triggers image acquisition (via the camera API of the Qt framework). In order to support a preselection of recently captured images, image previews are shown in a bar below and displayed in full resolution by tapping on them. Thereby, users can deselect erroneous images, and store the remaining ones either locally on the smartphone or in cloud storage.
- The gallery dialog presents a gallery view and enables the selection of images for image analysis.
- The settings dialog enables the configuration of camera focus and of the amount of subsequent images to be taken per recording. In addition, it enables the deletion of all locally stored images.

Since the development of the current application version focused on image-capturing and -processing functionality, the functionality for automatic reporting of results has yet to be implemented.

The Android application was developed for Android 6 (und upward) via C++ and the Qt QML 5.10 framework. The Android application integrates the components for image processing and classification.

#### 2.1.3. Image Processing

**Figure 6:**
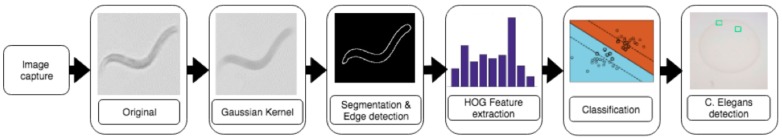
Applied image-processing toolchain for *C. elegans* detection.

For image processing, subsequent to image preprocessing, SVMs are used to train a classification model to detect *C. elegans* (as shown in Figure 6). Feature-representation method Histogram of Oriented Gradients (HOG) was used as feature representation due to its repeatedly reported good suitability to detect objects such as humans and writings in images [23,24,25].

By using backlight microscopy, the corresponding captured images experienced a strong contrast on the edges of *C. elegans*; that can be applied for automated classification via edge-detection-based approaches. However, this decision holds the risk that the edges of other objects, such as scratches or the border of the nutrient medium, would result in enhanced contrast. This must be handled by the preprocessing steps and the classification models.

Consequently, as a preprocessing step, a blurring Gaußian kernel [26] was applied to the (full) images to overcome such scratches and create uniform surfaces. Subsequently, the images were binarized via adaptive thresholding (with a maxval of 255, Open CV method adaptive_thresh_gaussian_C, a binary threshold type, a block_size of 101, and a subtracted constant from the mean of 3) and a Sobel operator with kernel size 1 [−1,0,1] was applied in the x and y directions for edge detection. The results are considered as the gradient vector of the HOG.

After preprocessing, the captured images are divided into overlapping region-of-interest (ROI) image sections with a size of 168 × 168 pixels per detection window. The detection window size is based on a variant of Dalal and Triggs, which successfully deployed a 64 × 128 pixel window size for human recognition [27]. Adjustment toward a square surface was based on the consideration that *C. elegans*, in contrast to humans, can take any orientation, and thus are thereby better covered. The 168 pixels fit common image sizes of smartphones as well (e.g., for the Samsung Galaxy S7 camera, the captured 3024 × 4032 pixels represent exactly 432 nonoverlapping ROI images per image). In order to assure the good coverage of *C. elegans*, a 50% overlap among ROI images in both width and height was applied.

Per ROI image, HOG features are calculated as follows: With g=gx2+g∗y2 and $\theta =arctan(g_{y}/g_{x})$, each gradient is characterized by grade g and direction θ, whereby $g_{x}$ and $g_{y}$ represent the gradients into the respective direction. Gradients are then combined into a dataset per each 8 × 8 pixelwide cell. Cellwise, these gradients are stored in a 9-bin histogram, wherein the 9 histogram classes represent the gradients’ direction as a 20 degree stepwise from 0 to 160 degree. Per cell, this pixelwise representation is added into the corresponding class of the 9-bin histogram. As a result, a 9-bin histogram vector is produced per cell.

Furthermore, block normalization was applied on the cellwise histogram vector. With a block consisting of 4 cells, the histograms of individual cells are merged to a vector with 36 elements that is normalized afterwards. Block normalization was conducted with an overlap of 50% in both dimensions and was expected to lower the effects of varying lighting conditions. Finally, the HOG-feature vector for each detection window was derived by concatenating all block vectors.

Based on the resulting HOG vectors, an SVM classification model was trained (as described in Section 3). For image-classification purposes, the SVM implementation of OpenCV 3.4 library was used with class-weight parameter C (representing the misclassification penalty) being set to 12.5, and the gamma of the Gaussian radial basis function being set to 0.5.

In addition, histogram intersection kernel $K\left(x_{i},x_{j}\right)=min\left(x_{i},x_{j}\right)$ was applied due to its comparably low computational complexity. It calculates the overlap between the histograms and thereby determines the fitting of two HOGs based on degree of fitting. Consequently, during classification, the SVM compares the fitting of the HOG vectors of the detection windows with the ones of the trained vectors of the training set.

### 2.2. Materials and Principle

For the evaluation study, *C. elegans* wild-type worms (N2, Caenorhabditis Genetics Center, Minneapolis, MN, USA) were cultivated at 20 °C on 8P agar plates seeded with *Escherichia coli* NA22, as previously described. For each experiment, eggs were picked with a 90% platinum, 10% iridium wire and seeded onto Nematode Growth Media (NGM) plates covered with the *E. coli* strain OP50 to obtain age-synchronous populations. The thin layer of the OP50 lawn facilitated the visualization of the transparent worms. L4-stage as well as adult worms were considered to clarify the development-stage independent suitability of the proposed microscope for detection.

The images were captured in this typical setting with the described smartphone-based microscope and LEDs being fully powered. Thereby, a Samsung Galaxy S7 was used and placed accordingly on the microscope holder. The Petri dishes were scanned with a closed lid. In this setting, 240 images were taken. For the conducted study, no ethics approval was required.

### 2.3. Methods

#### 2.3.1. Dataset Preparation

Since a *C. elegans* classification dataset is not yet available, we generated such with the introduced hardware setting and smartphone app.

**Figure 7:**
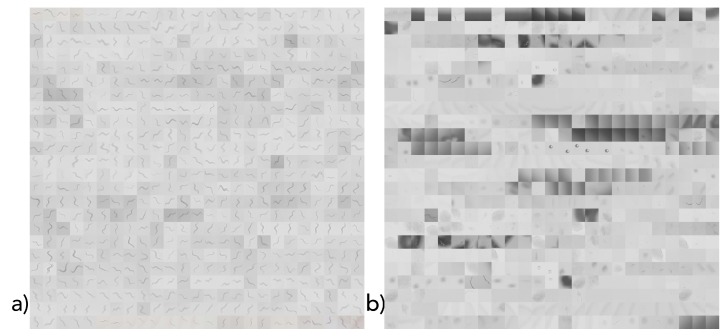
Resulting datasets of *C. elegans* covering (**a**) positive and (**b**) negative samples.

The dataset was manually created by selecting squares in the captured images that either contained a worm (positive) or did not (false). The corresponding images were then resized to 168 × 168 pixels images and joined into one positive and one negative image (as shown in Figure 7). In total, 1200 separate 168 × 168 pixel images (corresponding to their size, each with an image section) were created. Between these images, positive and negative samples were equally distributed. One-thousand equally distributed samples were applied as the training dataset, and 200 samples (100 positive and 100 negative) were used as the test dataset (shown in Figure 7). Since we used cross-validation, no separate development dataset was required.

#### 2.3.2. Training and Evaluation of Classification Model

For classification-model training, the two training images were rasterized with the detection window. Subsequently, the image preprocessing steps were applied and HOG features were calculated per detection window and stored into a vector. This vector was stored next to the corresponding label vector containing the corresponding classes of individual points in the corresponding order. SVM training via this vector pair was conducted via tenfold cross-validation.

The correspondingly applied algorithms are summarized in Table 1.

### 2.4. Evaluation of Computational Complexity of HOG-Feature-Based SVM on Full Images

The effect of the varying amount of *C. elegans* and window size on the classifiers’ computational complexity (in terms of processing time) was evaluated as follows.

For the tests, the system time at the following processing steps was recorded:

- Beginning;
- model loaded;
- HOG features of the windows are generated; and
- classification is concluded.

As the relevant processing steps, the duration of loading the model, generating HOG features, and classifying the results was considered. During evaluation, images were grouped according to the amount of given *C. elegans* per recorded Petri dish (up to 2, 13, and 25 worms). Ten images per group were considered. As 10 images per group were considered, the presented results show mean and standard deviation. In addition, we considered window sizes of 42 to 168 px.

In order to clarify the effect of the limiting processing power of smartphones (considered with the Samsung S7 smartphone), we ran processing on a common laptop computer as well, with an Intel i7 X 980 3.33 GHz CPU (with 6 kernels supporting up to 12 parallel threads), 12 GB RAM, a Samsung EVO 250 G SSD hard drive, and running 64-bit Ubuntu v.16.04.

## 3. Results

### 3.1. Similarity Evaluation of Dataset Element

In order to characterize the dataset, *p*-values were calculated for the HOG feature vectors of positive–negative sample pairs via the Python scikit-learn toolkit. From the resulting *p* values, the 13 NaN results are caused if the vector of the negative sample contained no elements. These special cases were interpreted as 0 *p*-values. With this necessary adjustment, the 600 *p*-values resulted in a mean of 0.0015, indicating the rejection of the null hypothesis that both sample pairs are equal. The rejection of the null hypothesis only failed in two cases (with maximal *p*-values of 0.05).

### 3.2. Evaluation of HOG-Feature-Based SVM in Test Dataset

In order to evaluate the sensitivity and specificity of the HOG-feature-based SVM, we examined it based on the test dataset. Thereby, the algorithm was evaluated with and without preprocessing. Results are summarized in Table 2 and indicate promising general sensitivity (of 0.90) and specificity (of 0.85).

The results indicate that the applied image-preprocessing steps represent a general benefit by increasing system sensitivity by 10%. Even though resulting in a decreased specificity of 10%, the general suitability of this preprocessing step is confirmed by the significant increase of around 0.01 for the F1 Score. With a general classification rate and an F1 Score of 87, the proposed prototype achieved a respectable classification rate for the automated classification of *C. elegans*

### 3.3. Evaluation of HOG-Feature-Based SVM on Full Images

**Figure 8:**
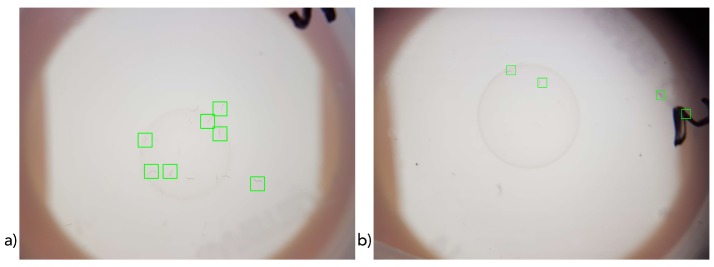
Classification results of example complete classified images. (**a**) Typical result and (**b**) extreme version containing typical low-specificity examples.

Besides the statistical evaluation of the classification rate, the classifier was qualitatively evaluated in complete images taken with the prototype to further investigate its general applicability. Thereby, further insights regarding the optimization of the system setup could be derived. The considered data represent full unprocessed images as taken by the smartphone camera within the developed hardware settings as processed by the final algorithm with image preprocessing and a 50% window overlap. In case of a true-positive classification, the considered area is framed in green. The corresponding classification results for the examples are shown in Figure 8. The cases of false-positive classification of nonworms in Figure 8b are representative for our quantitative experiments. Herein, the upper-right missclassification is the corner of adhesive tape; the lower-right is part of an inscription. Further, common errors of misclassification occurred very sporadically in cases of Petri dish scratches.

These images and our experiences with further examples confirm previously identified high sensitivity and prototype and algorithm specificity. *C. elegans* could be identified in most cases. Lack of sensitivity (in terms of missed *C. elegans* classifications) typically occurred mainly in cases of (multiple) overlapping worms. In addition, the windowing step width of 50% sometimes caused worm detection to be missed if they were not sufficiently covered by any window.

**Figure 9:**
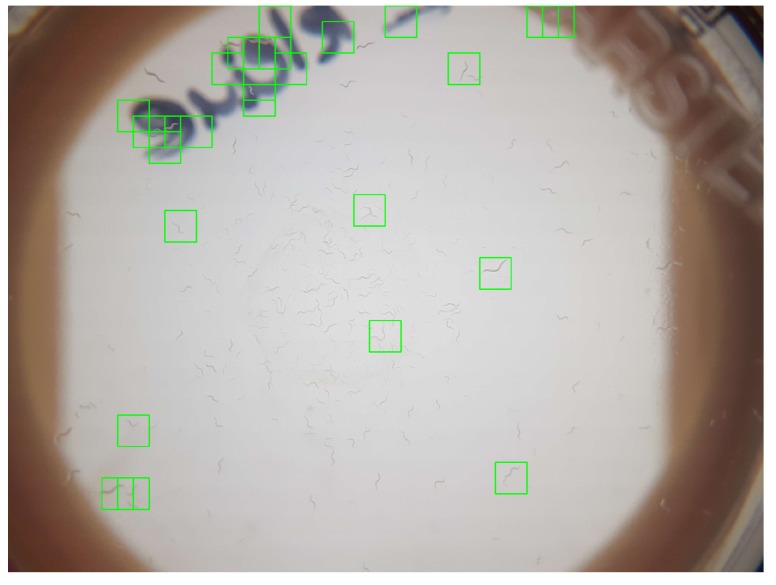
Classification results of complete classified images with unrealistic extreme *C. elegans* distribution density and age variations.

In order to investigate the limitations of the current ML algorithm, an extreme sample was qualitatively evaluated. By letting the *C. elegans* breed for multiple weeks, this Petri dish contained far more *C. elegans* (around 100) than practical, resulting in high distribution density. Furthermore, the *C. elegans* samples covered all developmental stages. While such a sample is unsuitable from an application standpoint, it is well-suited to clarify methodological limitations. In comparison with typical samples, the increased density of *C. elegans* resulted in their distribution over the complete Petri dish and increased the risk that multiple worms would overlap. As Figure 9 indicates, only a few *C. elegans* were correctly detected. While some false-positive detections occurred in the upper part due to the writing and the engraving of the Petri dish, most adult *C. elegans* could not be detected due to overlapping with other *C. elegans*. In addition, it should be pointed out that the current SVM model was only trained for adult *C. elegans*, which intentionally caused younger worms not to be detected. In addition, some of the separate *C. elegans* were not detected due to limited window sizes and the overlap, as discussed previously, which became necessary due to the limiting processing power of smartphones.

### 3.4. Evaluation of the Computational Complexity of HOG-Feature-Based SVM on Full Images

Furthermore, the effect of a varying amount of *C. elegans* and window size on the classifier’s computational complexity (in terms of processing time) was evaluated. Table 3 shows the corresponding results.

Since the duration of loading the classification model neither related to the amount of worms nor to the window size, it was not included in the table. Loading time only related to the device type, and took around 28 s on the smartphone and 1.65 s on the PC.

A dependency of processing times toward the device type was found for the generation of HOG features (HOG) and the classification step (Class.). On the smartphone, processing took typically three times longer than on the laptop.

Furthermore, the results in Table 3 clarify that neither for the HOG-feature-generation step nor for the Classification step was a dependency of processing time to the amount of *C. elegans* in the images given. Instead, window size clearly affects the processing time of both steps. For window sizes below 67 px, smartphone-based classification behaved unreliably, regularly leading to no classification. Consequently, a 42 window size could not be considered on the smartphone.

## 4. Discussion

With the aim to investigate if the image resolution of smartphone-camera sensors in combination with smartphones’ limited processing-power are sufficient for the automated detection of *C. elegans* for complete Petri dishes, we evaluated the sensitivity of an SVM-based classifier with images from the proposed hardware. Evaluation of the proposed system showed a classification sensitivity of 0.90 and a specificity of 0.85, thereby confirming its general practicability.

The cases of false-positive classification occurred due to adhesive tapes, inscriptions, or scratches. Since any of these three conditions can be omitted through corresponding standard operating procedures (SOPs), we are optimistic that specificity would be less critical in a controlled application. Such SOPs might require to remove to lid of the Petri dish that, in our case, remained attached. Due to the high visual similarity of the corner of adhesive tapes and *C. elegans*, and sufficient coverage via adjusted SOPs, we intentionally omitted a separate classification for them since we expected a general reduced classification rate.

The remaining challenges in regard to lack of sensitivity are a consequence of the 50% window overlap. While an increased window overlap (e.g., of 75%) can be expected to significantly lower this ratio, it can also be expected to increase computational complexity to a degree where processing is no longer applicable on a smartphone, as has been discussed for various window sizes). A corresponding evaluation of a 75% overlap already resulted in classification-processing times for a single image of over 30 s on a laptop computer. Thus, in our opinion, the presented configuration and resulting classification accuracy represent the practical limit if the smartphone-based classification of *C. elegans* via HOG features and an SVM are considered. Even though detection rate might be increased by lowering window sizes, the low processing power of smartphones imposes limits for the use of HOG-based SVMs. Consequently, we foresee the manual inspection and correction of missed *C. elegans* within the app to be a more suitable approach, and we will further investigate the suitability of alternative machine-learning methods.

With the sufficient sensitivity of *C. elegans* classification, further parameters (e.g., the average movement rate of the worms) can be derived besides the dose–response survival curve.

## 5. Conclusions

In conclusion, we introduced a smartphone-based microscope (including optics, lighting, and housing), and corresponding classification via a trained SVM for the automatic detection of *C. elegans* via a smartphone app. With a probability of 90% of correct recognition reached in the classification rates, and 87% in the F1 Score, the system achieved comparable sensitivity to the 93.4% of WorMachine, which utilizes a Olympus IX83 microscope using fluorescence excitation for image capture, and the 94% with a Leica confocal microscope by applying manual correction of foreground–background segmentation (81% without). Besides this promising quantitative result, qualitative evaluation within realistic images confirmed that, with the available means, satisfactory classification of *C. elegans* is possible. Correspondingly, our results indicate that the system sensitivity is comparable to that of other software solutions utilizing common combustion microscopes and could, thus, confirm the general practicability of the introduced approach. Due to its low pricing and higher degree of automation, the microscope is foreseen to be applicable for large-scale toxicity trials.

## Figures and Tables

**Table 1:** Statical-evaluation formulas.

Parameter	Formulars
True positive	*r_p_*
False negative	*f_n_*
True negative	*r_n_*
False positive	*f_p_*
Sensitivity	$\frac{r_{p}}{r_{p}+f_{n}}$
Specificity	$\frac{r_{n}}{r_{n}+f_{p}}$
False-negative ratio	$\frac{f_{n}}{r_{p}+f_{n}}$
False-positive ratio	$\frac{f_{p}}{r_{p}+f_{n}}$
Positive prediction value (PPV)	$\frac{r_{p}}{r_{p}+f_{p}}$
Negative prediction value (NPV)	$\frac{r_{n}}{r_{n}+f_{n}}$
Correct classification ratio	$\frac{r_{p}+r_{n}}{r_{p}+f_{p}+r_{n}+f_{n}}$
Misclassification ratio	$\frac{f_{p}+f_{n}}{r_{p}+f_{p}+r_{n}+f_{n}}$
F1 Score	$\frac{2\cdot (PPV\cdot Sensitivity)}{PPV+Sensitivity}$

**Table 2:** Statistical evaluation results of classification algorithm via test dataset.

Parameter	Result without Image Preprocessing	Result with Image Preprocessing
Sensitivity	0.80	0.90
Specificity	0.95	0.85
Positive prediction value	0.94	0.857
Negative prediction value	0.83	0.894
Correct classification rate	0.875	0.875
Misclassification rate	0.125	0.125
F1 Score	0.864	0.878

**Table 3:** Duration of processing steps (HOG: Generation of HOG features; Class.: Classification step) in seconds as mean and (SD) in relation to type of compute node, amount of worms per image and the applied window size (in px); Processing with a window size of 42 px on the smartphone resulted in system crash.

Type	Worms	Proc. Step	Window Size (in px)				
			42	67	84	126	168
Smartphone	2	HOG	-	17.07 (5.72)	10.79 (3.53)	4.80 (1.58)	2.70 (0.93)
	13	HOG	-	17.05 (5.67)	10.83 (3.68)	4.81 (1.68)	2.70 (0.92)
	25	HOG	-	16.98 (5.54)	10.73 (3.48)	4.78 (1.55)	2.67 (0.88)
	2	Class.	-	43.13 (14.31)	27.47 (10.65)	12.33 (4.23)	6.90 (2.68)
	13	Class.	-	43.77 (13.94)	26.94 (8.72)	12.18 (3.93)	6.94 (2.66)
	25	Class.	-	43.96 (16.07)	27.44 (9.49)	12.35 (3.98)	6.95 (2.25)
PC	2	HOG	14.73 (4.75)	5.84(2.04)	3.66 (1.19)	1.64 (0.55)	0.93 (0.32)
	13	HOG	14.53 (4.83)	5.76 (1.90)	3.65 (1.22)	1.66 (0.54)	0.92 (0.31)
	25	HOG	14.52 (4.83)	5.83 (1.95)	3.66 (1.19)	1.63 (0.55)	0.92 (0.31)
	2	Class.	32.03 (10.68)	12.81 (4.22)	8.15 (2.75)	3.72 (1.21)	2.13 (0.70)
	13	Class.	31.92 (10.57)	12.78 (4.25)	8.10 (2.69)	3.70 (1.22)	2.11 (0.70)
	25	Class.	31.88 (10.60)	12.74 (4.25)	8.12 (2.70)	3.68 (1.24)	2.10 (0.71)
